# Hexagonal Boron Nitride Functionalized with Au Nanoparticles—Properties and Potential Biological Applications

**DOI:** 10.3390/nano8080605

**Published:** 2018-08-09

**Authors:** Magdalena Jedrzejczak-Silicka, Martyna Trukawka, Mateusz Dudziak, Katarzyna Piotrowska, Ewa Mijowska

**Affiliations:** 1 Laboratory of Cytogenetics, West Pomeranian University of Technology, Szczecin, Klemensa Janickiego 29, 71-270 Szczecin, Poland; 2 Nanomaterials Physicochemistry Department, West Pomeranian University of Technology, Szczecin, Piastow Avenue 45, 70-311 Szczecin, Poland; martyna.brylak@zut.edu.pl (M.T.); mateusz.dudziak@zut.edu.pl (M.D.); 3 Department of Physiology, Pomeranian Medical University in Szczecin, Powstancow Wlkp. 72, 70-111 Szczecin, Poland; piot.kata@gmail.com

**Keywords:** boron nitride, nanocomposite, functionalization, gold nanoparticles, cytobiocompatibility

## Abstract

Hexagonal boron nitride is often referred to as white graphene. This is a 2D layered material, with a structure similar to graphene. It has gained many applications in cosmetics, dental cements, ceramics etc. Hexagonal boron nitride is also used in medicine, as a drug carrier similar as graphene or graphene oxide. Here we report that this material can be exfoliated in two steps: chemical treatment (via modified Hummers method) followed by the sonication treatment. Afterwards, the surface of the obtained material can be efficiently functionalized with gold nanoparticles. The mitochondrial activity was not affected in L929 and MCF-7 cell line cultures during 24-h incubation, whereas longer incubation (for 48, and 72 h) with this nanocomposite affected the cellular metabolism. Lysosome functionality, analyzed using the NR uptake assay, was also reduced in both cell lines. Interestingly, the rate of MCF-7 cell proliferation was reduced when exposed to h-BN loaded with gold nanoparticles. It is believed that h-BN nanocomposite with gold nanoparticles is an attractive material for cancer drug delivery and photodynamic therapy in cancer killing.

## 1. Introduction

Recently, graphene, related two-dimensional crystals and hybrid particles have been intensively studied in the context of technological and scientific evolution. Unique properties of graphene allow for using it for various purposes in many fields (e.g., biomedical applications, energy storage, electronic devices, biosensors, spintronics or photonics) [1]. Hexagonal boron nitride (h-BN) is one of the 2D layered materials with specific properties [1]. BNs were considered only as synthetics, but recently they have also been discovered in the natural environment (Qingsongite (IMA2013-30)—natural analogue of cubic boron nitride) [2,3]. Hexagonal boron nitride is an analogue of graphite. In its structure, alternating B and N atoms substitute C atoms [4]. Boron and nitrogen atoms are linked with each other via strong B-N covalent bonds to form interlocking hexagonal rings [2,4]. The 2D layers of h-BN are held together by weak van der Waals forces [2,5,6]. The B-N bond length is 1.466 Å, whereas the interlayer space is 3.331 Å [5]. In comparison to graphene, where C-C are covalently bonded, h-BN covalent B-N bonds are partially ionic; this is due to B atoms, which in every consecutive BN layer are positioned exactly above or below N atoms in the adjacent layers. Such structural characteristics implies the polarity of B-N bonds [2,4]. Boron nitride can also exhibit a hollow spherical morphology [4,7], diamond-like cubic form (c-BN), with boron and nitrogen atoms forming a tetrahedral bond network, as well as wurtzite BN (w-BN). The cubic structure with alternating boron and nitrogen atoms is similar to that of diamond [5]. Its flakes may be mono- or several-layer-thick. The formation of multilayer stabilizes the whole structure. h-BN systems (e.g., nanotubes, flakes) show chemical and thermal stability, but at the same time they are equally thermally conductive and mechanically robust. h-BN is an electrical insulator with a band gap of ~5–6 eV [4].

h-BN is used in different fields due to its interesting physical and chemical properties, e.g., in electronics as an insulator, substrate for semi-conductors, coating for refractory molds, in ceramics, resins, plastics (to obtain self-lubricating properties) [5,7,8]. BN nanosheets were found useful in polymeric film reinforcement, e.g., the elastic modulus of polymethyl methacrylate (PMMA) film was increased when BN nanosheets were incorporated into the polymer [4]. Hexagonal boron nitride is also widely used in the production of coatings and paintings for high temperature applications. Boron nitride is also a popular inorganic compound in cosmetic industry used as a slip modifier [5,7]. Lately, hexagonal boron nitride has been found to be excellent substrate material [9,10]. The h-BN platforms are used in creation of a new generation of few-atomic-layer vdW (van der Waals) heterostructures. Most 2D heterostructures (vertical heterostructures) are synthesized by stacking of individual layers of different materials [10,11]. Unlimited van der Waals interplanar interactions in layered materials provide possibility to integrate with an array any layered 2D material (such as graphene, hexagonal boron nitride, transition-metal dichalcogenides) of different dimensionality (e.g., combinations of 2D + *n*D materials, where *n* = 0, 1 and 3) [12]. Zhao and co-workers (2018) presented controlled electrochemical intercalation of graphene/h-BN vdW heterostructures, where Li was electrochemical intercalated into graphene encapsulated between h-BN layers resulting in higher carrier density [9,13]. Using of exfoliated h-BN crystals [14] as a substrate, crystalline high-quality rubene have been template. Obtained heterostructure enabling creation of organic FETs (OFETs) with carrier motilities exceeding 10 cm^2^ V^−1^ s^−1^ [12,14,15]. By comparison, graphene on ultra-flat boron nitride (BN) has shown intrinsic mobility approaching 500,000 cm^2^ V^−1^ s^−1^ [16]. Those structures display unique properties, diverse functionality, great potential and can response to the need of electronic and electrochemical industry [13]. Due to their ultrasensitivity, 2D heterostructures present a broad range of applications, e.g., photovoltaic [12], field effect/tunnelling transistors, optronics [17], photodetectors, light-emitting [18], electronic [19], thermoelectric and memory [15,19] devices and bio-sensing [20]. 

Boron nitride exhibits also hydrophobicity in aqueous environment [21]. Therefore, it seems to be suitable for biomedical applications after specific functionalization process. The problem of limited BNs dispersion is one of the most challenging approaches [22]. Several cytotoxicity studies based on boron nitride nanotubes (BNNT), hollow boron nitride nanospheres, h-BN nanosheets confirmed its low cytotoxicity and suggested that BN can be used as a novel drug delivery system. In contrast, other studies have showed that BNNT had cytotoxic effect and affected relative cell viability even at low concentrations [23,24,25,26].

Although boron nitrides have unique and applicable properties, the number of BN-related publications is significantly smaller in comparison to the widely studied C systems [3]. Thus the aim of the study was to evaluate exfoliated hexagonal boron nitride functionalized with Au nanoparticles, for potential biomedical applications.

## 2. Materials and Methods

### 2.1. Materials

Hexagonal boron nitride, gold(III) chloride trihydrate, phosphate buffered saline, polyethylene glycol, Pluronic F-127, dehydrate trisodium citrate were purchased from Sigma-Aldrich (St. Louis, MO, USA). Hydrogen peroxide solution, sulfuric acid, potassium permanganate and 1-methyl-2-pyrrolidinone were obtained from Chempur (Piekary Slaskie, Poland).

### 2.2. Methods

#### 2.2.1. Exfoliation of h-BN

Chemical exfoliation of h-BN was carried out by a modified Hummer’s method, similar to graphite exfoliation. 750 mg of h-BN was mixed with 3.0 g of potassium permanganate in a three-neck flask. The whole system was installed under the reflux. Next, 60 mL of 96% sulfuric acid was slowly added. The mixture was heated at 40 °C for 6 h. Subsequently, the system was cooled down. The flask with the mixture was inserted into the ice bath. Then 200 mL of hydrogen peroxide solution was added. After this process, the mixture was purified. Purification was carried out via multiple water washing and centrifugation at 10,000 rpm for 15 min until the pH reached 7.

Chemically exfoliated h-BN was additionally delaminated mechanically. Mechanical exfoliation was performed using a tip sonicator. Chemically exfoliated h-BN was added into 1-methyl-2-pyrrolidinone (NMP) in a volume ratio of 0.5% and sonicated (600 W 25%) for 30 min with a pulse mode of 5s on/5s off. After sonication, the mixture was left to evaporate the solvent.

#### 2.2.2. Hexagonal Boron Nitride Au Functionalization

Exfoliated h-BN was functionalized with gold nanoparticles. 100 mL of distilled water was mixed with 6 mg of h-BN. The mixture was heated at 100 °C under the reflux. Next 4 mL of gold(III) chloride trihydrate was added at a concentration of 2 mg mL^−1^. After a few minutes, 40 mg of trisodium citrate was added to the boiling content. The whole system was heated for 1 h at 100 °C. After 1 h, the mixture was cooled down for the purification. The purification was performed by multiple washing with distilled water and centrifugation at 8000 rpm for 10 min until the pH reached 7.

### 2.3. Characterization of Synthesized Nanomaterial

The samples were examined using transmission electron microscopy (TEM, FEI Tecnai F30, Frequency Electronics Inc., Thermo Fisher Scientific, Waltham, MA, USA). The phase composition of samples was characterized by X-ray diffraction (XRD) analysis (X’Pert PRO Philips diffractometer, Almelo, The Netherlands) using a CoK_α_ radiation. UV-Vis absorption spectra of h-BN, h-BN nanocomposite (h-BN_AuNP) and gold nanoparticles were recorded with a Helios alpha UV-Vis Spectrometer (Thermo Fisher Scientific, Waltham, MA, USA). Fourier transform infrared (FT-IR) absorption spectra were measured on a Nicolet 6700 FT-IR spectrometer (Thermo Nicolet Corp., Madison, WI, USA). Raman spectra were measured with a Renishaw in via Raman microscope at 785 nm. The surface and thickness of the flakes were measured by atomic force microscopy (AFM, Nanoscope V Multimode 8, Bruker, Mannheim, Germany). Zeta potential was measured by Zeta Sizer (ZS Nano, Malvern Panalytical, Malvern, UK).

### 2.4. Dispersion Stability of Au Functionalized h-BN

Before cytocompatibility analyses, the dispersion stability of hexagonal boron nitride was examined in phosphate buffered saline (PBS) with dispersant Pluronic F-127. Concentration of PBS-Pluronic F-127 was 1 mg mL^−1^. Subsequently, different amounts of nanomaterial (12.5 µg mL^−1^, 25 µg mL^−1^, 50 µg mL^−1^ and 100 µg mL^−1^, respectively) was diluted with PBS-polymer and sonicated to obtain homogeneous solution of the following concentration. The UV-Vis monitoring (Thermo Scientific GENESYS 10S, Thermo Fisher Scientific, Waltham, MA, USA) at 350 nm was evaluated to determine the dispersions stability after 1, 3, 5, 20, 22, 24, 44, 48 and 51 h.

### 2.5. Cell Lines and Cell Culture Conditions

Two adherent cell lines—murine L929 fibroblast (ATCC^®^ CCL-1™, American Type Culture Collection, Manassas, VA, USA) and MCF-7 human breast adenocarcinoma (ATCC^®^ HTB-22™, American Type Culture Collection, Manassas, VA, USA)—were chosen for cytocompatibility studies of the h-BN_AuNP nanocomposite.

For morphology analyses (phase contrast and holographic microscopy), cells of each line were seeded into T25 flasks (Sarstedt, Nümbrecht, Germany) and maintained in standard cell culture conditions at 37 °C, 5% CO_2_, 95% humidity. Complete Dulbecco’s Modified Eagle Medium (DMEM) culture medium supplemented with 10% heat inactivated fetal bovine serum (FBS) (PAA Laboratories GmbH, Pasching, Austria), 2 mM l-glutamine, 50 IU mL^−1^ penicillin and 50 µg mL^−1^ streptomycin (Sigma-Aldrich, St. Louis, MO, USA) was used in the study.

For cytocompatibility analysis, the cells were seeded into 96-well plates (Corning Inc., New York, NY, USA) and cultured for 24, 48 and 72 h in standard conditions mentioned above. All cell cultures were monitored with a Nikon TS-100 microscope (Nikon, Melville, NY, USA).

### 2.6. Experimental Treatment

For experimental treatment, the h-BN_AuNP nanocomposite was prepared in a 100 µg mL^−1^ Pluronic F-127 solution in PBS to a stock concentration of 1 mg mL^−1^. Twenty-four hours after cell seeding, 8 different final concentrations (3.125, 6.25, 10.0, 12.5, 25.0, 50.0, 100.0, 200.0 µg mL^−1^) of h-BN_AuNP nanocomposite were prepared in DMEM medium and added to the cell cultures. Additionally, the Pluronic F-127 vehicle control (only DMEM medium with dispersant) was prepared for the reference dispersion as well as the standard control culture (cells cultured in standard DMEM medium in the absence of the h-BN_AuNP nanocomposite and Pluronic F-127). Cell lines were incubated with the h-BN_AuNP nanocomposite for 24, 48 and 72 h.

### 2.7. Microscopic Analyses

Firstly, the morphology of L929 and MCF-7 cell lines exposed to the h-BN_AuNP nanocomposite at different concentrations and control samples was analyzed using a Nikon TS-100 phase contrast inverted microscope (Nikon, Melville, NY, USA) at 400× magnification.

Secondly, holographic microscopy label-free images were obtained using the HM4 HoloMonitor™ (Phase Holographic Imaging, Lund, Sweden). For presented analyses, the HoloMonitor™ connected to the computer was placed inside a CO_2_ incubator (Memmert GmbH, Germany) to capture time-lapse image sequences during the cell treatment. Similarly to the phase contrast microscopy, the images using the HoloMonitor M4 were taken for different concentrations of the h-BN_AuNP nanocomposite and the control sample for both cell lines. The time-laps image sequence was recorded every 1 min for 24–72 h (24 h after cell seeding). The doubling time (DT) was established basing on holographic observations, as a measure of cell growth for each cell line using the following Formula (1):

(1) $$\mathrm{Doubling}\;\mathrm{Time}=\frac{\mathrm{duration}\times \log(2)}{\log\left(\mathrm{Final}\;\mathrm{concentration}\right)-\log(\mathrm{Initial}\;\mathrm{concentration})}\;$$

### 2.8. Cell Counting Kit-8 Analysis

The relative mitochondrial activity of L929 and MCF-7 cell lines after 24, 48 and 72-h incubation with the h-BN_AuNP nanocomposite was tested using the CCK-8 Cell Counting Kit-8 (Sigma-Aldrich, St. Louis, MO, USA). The CCK-8 assay is based on the conversion of tetrazolium salt into the colored formazan by the living cells. The amount of the reduction product is proportional to the number of metabolically active cells. The CCK-8 solution (10 µL) was added to each well and incubated for 240 min at 37 °C in an incubator. After the incubation period, the absorbance was recorded at 450 nm, according to the manufacturer’s instructions using a Sunrise Absorbance Reader (Tecan, Männedorf, Switzerland). All experiments were conducted in triplicate.

The effect of the nanocomposite on cellular metabolic activity was calculated using the following Formula (2):

(2) $$\mathrm{Relative}\;\mathrm{viability}\;\mathrm{from}\;\mathrm{the}\;\mathrm{CCK}-8\;\mathrm{assay}(\%)=\left(\frac{{\mathrm{sample}\;\mathrm{abs}}_{450–650\;\mathrm{nm}}}{{\mathrm{positive}\;\mathrm{control}\;\mathrm{abs}}_{450–650\;\mathrm{nm}}}\right)\times 100$$

### 2.9. Lactate Dehydrogenase Leaking Assay

Cytocompatibility of the h-BN_AuNP nanocomposite was evaluated using the LDH CytoTox 96^®^ Non-Radioactive Cytotoxicity Assay (Promega, Madison, WI, USA). The LDH CytoTox 96^®^ Non-Radioactive Cytotoxicity Assay measures lactate dehydrogenase released due to cellular membrane damage. The amount of formazan converted from tetrazolium salt is proportional to the number of lysed cells. The LDH assay was performed according to the manufacturer’s instructions (Promega, Madison, WI, USA) and the absorbance was measured at 490 nm using a microplate spectrophotometer (Absorbance Reader, Tecan, Männedorf, Switzerland). The interaction between the solution with the nanocomposite in the cell culture medium and LDH assay components was tested in the absence of cells. The percentage of LDH released after 24, 48 and 72-h exposure was calculated using the Formula (3):

(3) $$\%\mathrm{LDH}\;\mathrm{released}=\frac{A490\;\mathrm{nm}\;\mathrm{of}\;\mathrm{treated}\;\mathrm{and}\;\mathrm{untreated}\;\mathrm{cells}-A490\;\mathrm{nm}\;\mathrm{of}\;\mathrm{control}}{A490\;\mathrm{nm}\;\mathrm{of}\;\mathrm{maximum}\;\mathrm{of}\;\mathrm{untreated}\;\mathrm{cells}-A490\;\mathrm{nm}\;\mathrm{of}\;\mathrm{control}}\times 100$$

where *A* is absorbance.

### 2.10. Neutral Red Uptake Assay

Similarly, the neutral red uptake assay (NRU) (In vitro Toxicology Assay Kit, Neutral Red based, Sigma-Aldrich, St. Louis, MO, USA) was performed 24–72 h after the L929 and MCF-7 cell exposure to the tested nanocomposite. The neutral red uptake assay is based on the viable cell ability to store the neutral red dye in acidic organelles by the active transport (e.g., in lysosomes). Fresh DMEM medium containing 10% of neutral red was added to the cultures and incubated at 37 °C, 5% CO_2_ and 95% relative humidity for 3 h. After the incubation, the cells were washed with DPBS and the Solubilization Solution was added to release the incorporated dye from the cells. The culture plates were allowed to rest for 10 min at room temperature, then gentle stirred and the absorbance at 540 nm was measured using a Tecan Sunrise microplate reader (Tecan, Männedorf, Switzerland). The effect of the nanocomposite on cell viability was calculated using the following Equation (4):

(4) $$\mathrm{Neutral}\;\mathrm{red}\;\mathrm{uptake}\;\mathrm{assay}(\%)=\left(\frac{{\mathrm{sample}\;\mathrm{abs}}_{540–690\;\mathrm{nm}}}{{\mathrm{positive}\;\mathrm{control}\;\mathrm{abs}}_{540–690\;\mathrm{nm}}}\right)\times 100$$

### 2.11. Cellular Uptake and Confocal Microscope Imaging

For the cellular uptake analysis, cells were plated in chamber slides (Lab-Tek Chamber, 4-wells, Thermo Scientific, Waltham, MA, USA) and cultured for 24 h. 24 h after the cell seeding, 50 µg mL^−1^ h-BN nanoflakes labeled with FITC (Sigma-Aldrich, St. Louis, MO, USA) were added to the culture medium and incubated for additional 24–72 h. Afterwards, the cells were washed three times with DPBS (PAN-Biotech GmbH, Aidenbach, Germany) and they were fixed with 4% paraformaldehyde solution (Sigma-Aldrich, St. Louis, MO, USA) for 10 min at RT. Next, for the cellular nuclei localization cells were stained with DAPI solution (5 µg mL^−1^, Sigma-Aldrich, St. Louis, MO, USA) for 20 min at RT. The microphotographs were collected in FV1000 Confocal system with Olympus IX81 inverted microscope (Olympus, Hamburg, Germany) in two separated channels: for DAPI (405 nm diode), for FITC (488 nm laser). The microphotographs are showed as merged image.

### 2.12. Statistical Analysis

Data collected in CCK-8, LDH, and NRU assays are given as the mean values ± standard deviation (SD) and analyzed using ANOVA. The statistical analyses for CCK-8 and LDH assay results were performed using Levene’s test of homogeneity. For data obtained from NRU assays Kruskal-Wallis analyses were conducted. The *p*-values < 0.05 were considered significant and are represented by different small letters (Figure 8A–F). Statistical analyses were performed using the *STATISTICA* 12.5 (StatSoft Inc., Tulsa, OK, USA) software.

## 3. Results

### 3.1. Characterization of h-BN and h-BN_AuNP

**Figure 1:**
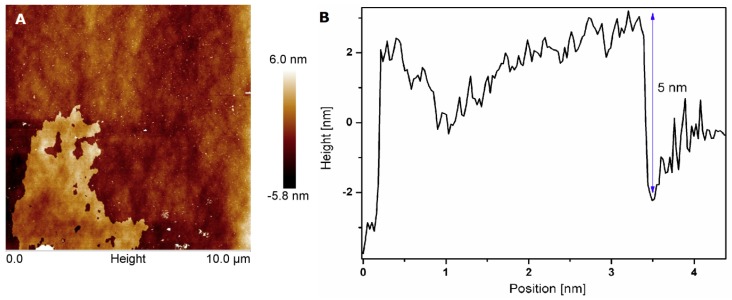
Atomic force microscope analysis for surface morphology (**A**) and flake thickness (**B**) of exfoliated h-BN.

The morphology of h-BN was evaluated using transmission electron microscopy. The most important was to assess the shape and thickness of h-BN flakes before and after the exfoliation process. The commercial h-BN flakes were very thick (~300 nm) [27,28], which corresponded to 900 layers in the individual flake. A large number of flakes was aggregated and connected to each other. After combining chemical and mechanical exfoliation methods, thin flakes have been obtained (shown in Figure S1A, Supplementary materials). Atomic force microscopic analyses also confirmed this feature of h-BN. By using AFM, it is possible to calculate thickness of a single h-BN flake. Thickness of bulk h-BN is about 166 layers in individual flake. The morphology and thickness of exfoliated h-BN is shown in Figure 1A,B. Based on our calculations, the flake thickness after exfoliations was about 5 nm.

**Figure 2:**
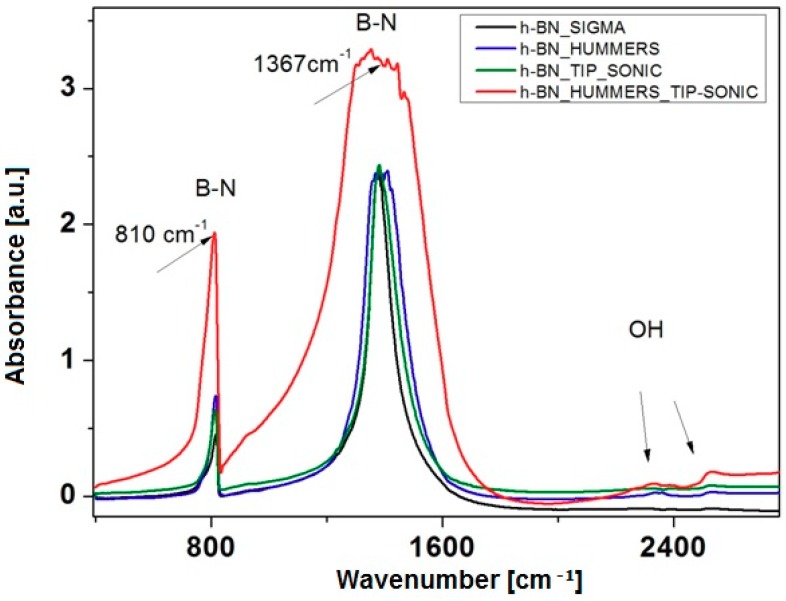
FT-IR spectra of bulk and exfoliated h-BN.

In order to analyze the presence of the functional groups induced in the exfoliation process, FT-IR spectra were obtained for commercial h-BN and h-BN exfoliated by combined methods (chemical and physical exfoliation). Figure 2 shows the differences in spectra between bulk and exfoliated h-BN. No functional groups were found in commercial h-BN. O-H groups appeared after chemical exfoliation, with peaks near 2526 and 2330 cm^−1^. The peaks at 1367 cm^−1^ and 810 cm^−1^ are derived from hexagonal boron nitride and represented B-N bonds.

To confirm the interactions of gold nanoparticles and h-BN, a Fourier Transform Infrared Spectroscopy (FTIR) study was conducted. The spectra of h-BN and h-BN_Au are compared in Figure S2 (Supplementary materials). There are three characteristic peaks in h-BN. The peak at 810 cm^−1^ corresponds to B-N vibrations. And the band at 940 cm^−1^ is assigned to B-N-O [29]. The peak at 1370 cm^−1^ is due to the stretching vibrations of B-N. The hydroxyl band was observed at 2525 and 3400 cm^−1^ for h-BN. After the Au functionalization these peaks disappeared. Interestingly, it is noteworthy that the sample of h-BN with Au shows a new band near 1100 cm^−1^, which corresponds in particular to C–O stretching vibrations [30]. That is, when Au^3+^ ions are reduced with the hydroxyl groups of the boron nitride, the hydroxyl groups are oxidized to carbonyl group. It can be concluded from the above results that Au^3+^ ions can be reduced by hydroxyl groups of h-BN, resulting in the formation of gold nanoparticles on the surface [31]. It is also worth mentioning that the peak at 1370 cm^−1^ changed its shape and width, what can also confirm the interactions between h-BN and gold nanoparticles.

**Figure 3:**
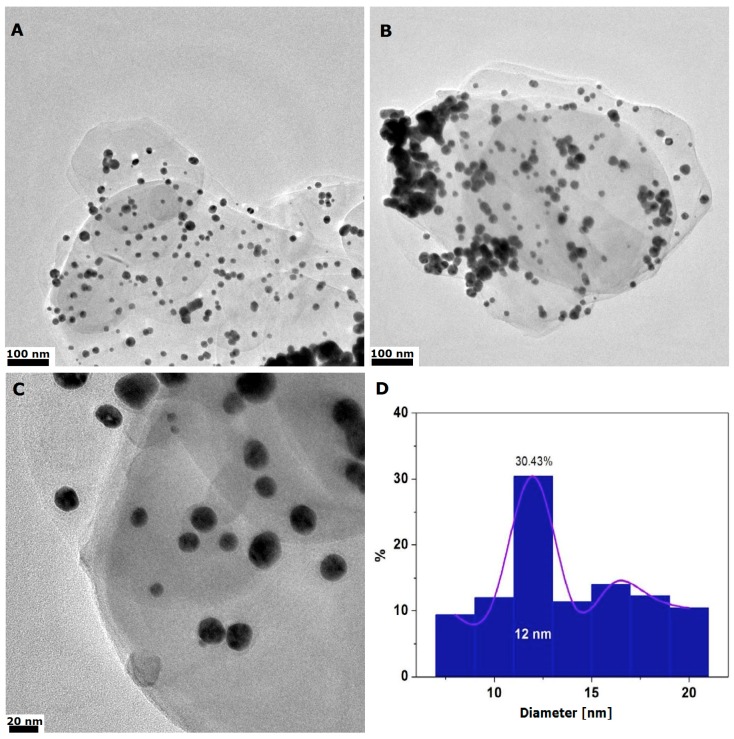
Transmission electron microscope images of h-BN_AuNP nanocomposite (**A**–**C**) and histogram of particle size distribution (**D**).

Morphology and thickness of h-BN_AuNP nanocomposite is shown in Figure 3. Transmission electron microscopy confirmed that gold nanoparticles were deposited on the h-BN surface. The size of gold particles ranged from 10 to 20 nm with the average value of 12 nm (about 30.5%; Figure 3D). There was not many agglomerates of gold nanoparticles. Most of nanoparticles were homogeneously distributed on h-BN platform. Thickness of the nanocomposite was similar to the exfoliated h-BN. The morphology of h-BN_AuNP is shown in Figure 3.

**Figure 4:**
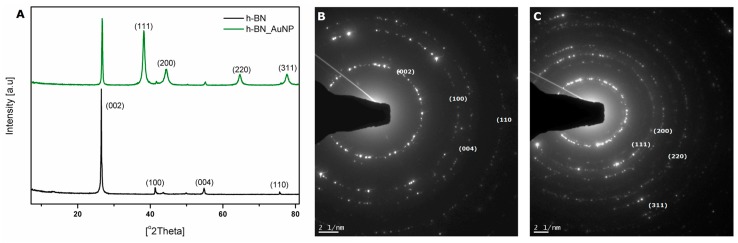
XRD patterns (**A**), electron diffraction patterns of h-BN (**B**) and h-BN_AuNP (**C**).

The XRD analysis and selected area electron diffraction pattern of the h-BN exfoliated and h-BN_AuNP nanocomposite are shown in Figure 4A. On h-BN XRD pattern there are four peaks at the Bragg angles of 26.52 2Θ; 41.3 2Θ; 54.8 2Θ; 76 2Θ, that correspond to (002), (100), (004) and (110) planes of h-BN, respectively. These diffraction peaks prove the hexagonal structure of boron nitride [32].

With regard to the h-BN_AuNP nanocomposites, Bragg angles of 38.3 2Θ; 44.3 2Θ; 64.5 2Θ and 77.6 2Θ were clearly observed, which correspond to the (111), (200), (220) and (311) planes of the AuNPs. There are also characteristic diffraction peaks coming from h-BN. These results confirmed that the AuNPs anchored on h-BN were well crystallized. The AuNPs/h-BN nanocomposite was successfully prepared [33]. The electron diffractions pattern (Figure 4B,C) confirms the results obtained from X-ray diffractometer.

**Figure 5:**
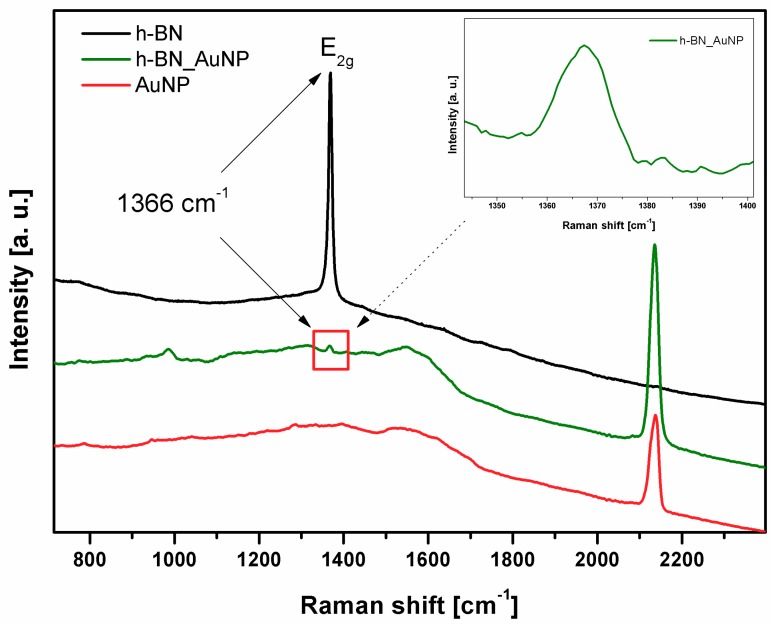
Raman spectra of h-BN, h-BN_AuNP nanocomposite and AuNP.

Raman spectra (Figure 5) were measured for 3 samples: exfoliated h-BN, h-BN_AuNP nanocomposite and gold nanoparticles. The most characteristic peak in h-BN at 1366 cm^−1^ is due to the E2g phonon mode and analogous to the G peak in graphene [4,10,34,35]. It can be seen in the spectra of h-BN and Au nanocomposite spectra. The peak is very intensive in h-BN spectrum. Its intensity is much smaller in the nanocomposite due to presence of gold nanoparticles—they have characteristic peaks in the region from 1300 to 1600 cm^−1^. Similar observations were made by Draz et al. (2016) in their research [36]. However, the most prominent peak confirming the functionalization of the h-BN by gold nanoparticle is at about 2130 cm^−1^. It is present in the spectrum of pristine gold nanoparticles and the nanocomposite with h-BN. 

**Figure 6:**
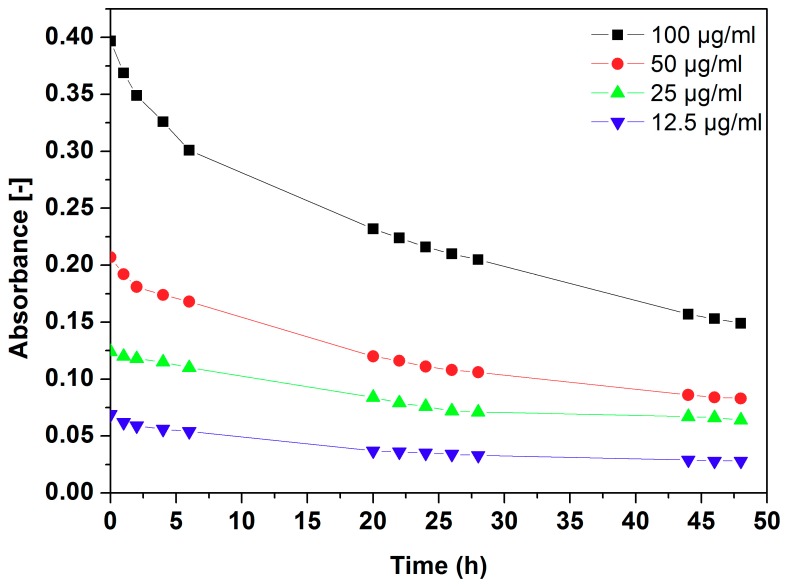
Dispersion stability of h-BN_AuNP with nanomaterial concentration range of 12.5–100 µg mL^−1^.

The stability of h-BN_AuNP nanocomposite is shown in Figure 6. A UV-Vis spectrophotometer was used for its evaluation. The solution was prepared by the dissolving one tablet of PBS in 200 mL of water. Next, the solution of Pluronic F-127 100 µg mL^−1^ was prepared. The h-BN_AuNP nanocomposite was added to the polymer solution in PBS to the final concentrations of 12.5, 25, 50 and 100 µg mL^−1^, respectively. The dispersion stability was verified for 48 h. The best results were obtained at the concentration of 12.5 µg mL^−1^. However, the dispersion was stable also at higher concentrations. 

In order to determine what the loading of gold in the nanocomposite, the UV-Vis spectrophotometry was used. The peak near 530 nm indicated that there were particles about 10 nm in size. Figure S3A (Supplementary materials) represents spectrum of pristine h-BN showing only one peak ~200 nm, while the h-BN_AuNP nanocomposite exhibited also a peak at 530 nm. The simple quantitative analysis allowed to conclude that there was ~16 wt% of the nanocomposite.

The UV-Vis studies were also performed to determine the stability of the interaction between hexagonal boron nitride and gold nanoparticles. At appropriate time intervals, the nanocomposite solution was tested on a UV-Vis spectrometer to determine the possible changes in the concentration of gold in the solution. The experiment was carried out for 6 h in distilled water. The results indicate that gold was not released to the solution. Therefore, it can be assumed that the deposition of Au on hexagonal boron nitride is stable (Figure S3B, Supplementary materials).

Zeta potential was measured for h-BN and for nanocomposite. The particles with values of zeta potential between +20 and −20 mV are considered rather unstable. Particles with values more positive than +20 mV and more negative than −20 mV are normally considered stable [37]. Both the nanocomposite and the pristine material showed a negative value of zeta potential, with values of −27.6 ± 5.95 mV, and −29.7 ± 6.81, respectively.

### 3.2. In Vitro Microscopic Analyses

**Figure 7:**
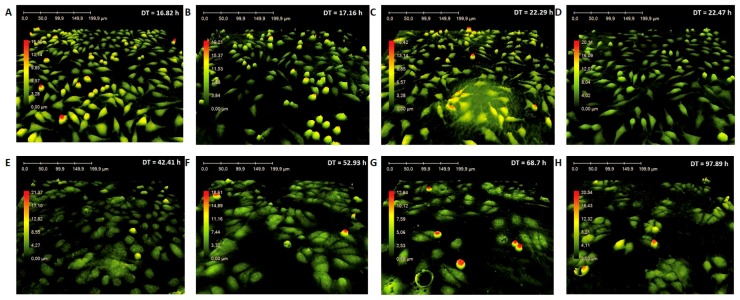
Morphology of the L929 and the MCF-7 cell lines incubated with the h-BN_AuNP nanocomposite. L929 control culture (**A**), L929 culture at 24 h (**B**), 48 h (**C**) and 72 h (**D**), MCF-7 control culture (**E**), MCF-7 culture at 24 h (**F**), 48 h (**G**) and 72 h (**H**) (DT—doubling time).

Microscopic observations were carried out after 12-h incubation to record the effect of the h-BN_AuNP nanocomposite on both cell lines. As shown in Figure 7, the cells observed under a light microscope exposed to the nanocomposite at a concentration of 10.0 µg mL^−1^ exhibited visible differences in comparison to the control culture (Figure S4A,C, Supplementary materials). In the case of L929 cell line, h-BN_AuNP affected the cellular membrane. Numerous small membrane vesicles were observed in L929 cells. The shape of L929 cells was not changed and the cells showed no tendency for detachment (Figure 7A). In contrast, MCF-7 cells did not exhibit evident changes in the shape and membrane vesicles (Figure 7B). The effect of h-BN_AuNP nanocomposite in MFC-7 cells could be observed by the presence of vacuoles in the cell cytoplasm. Lower cell count was also found in the experimental MCF-7 culture in comparison to the MCF-7 control culture (Figure 7E).

Similarly, time-lapse image sequences were taken using a HoloMonitor™ M4. L929 and MCF-7 cell lines were exposed to the h-BN_Au nanocomposite at a concentration of 10.0 µg mL^−1^ for 24, 48 and 72 h (Figure 7). L929 cells did not show any significant differences in the presence of the nanocomposite (Figure 7B–D). The DT value was determined basing on image sequences. The DT value for the L929 control was 16.82 h, while the DT values of the experimental culture were 17.16 h, 22.29 h and 22.47 h for 24-, 48- and 72-h incubation, respectively. L929 cells showed no reduction in proliferation under experimental conditions for cultures exposed to h-BN_AuNP during a 24-h incubation, whereas cell doubling times for 48- and 72-h incubations were higher in comparison to the control culture. 

Results obtained for the MCF-7 cell line incubated with h-BN_AuNP demonstrated a stronger effect on the cells at a concentration of 10.0 µg mL^−1^ (Figure 7F–H). MCF-7 cells did not show any visible morphological changes in comparison to the control culture, but the DT analysis indicated a reduction in proliferation capacity. The DT value for the MCF-7 control sample was 42.41 h, whereas the doubling time for experimental cultures was 52.93 h, 68.70 h, and 97.89 h for 24-, 48-, and 72-h incubations, respectively. 

### 3.3. Analysis of Cytotoxicity Results

**Figure 8:**
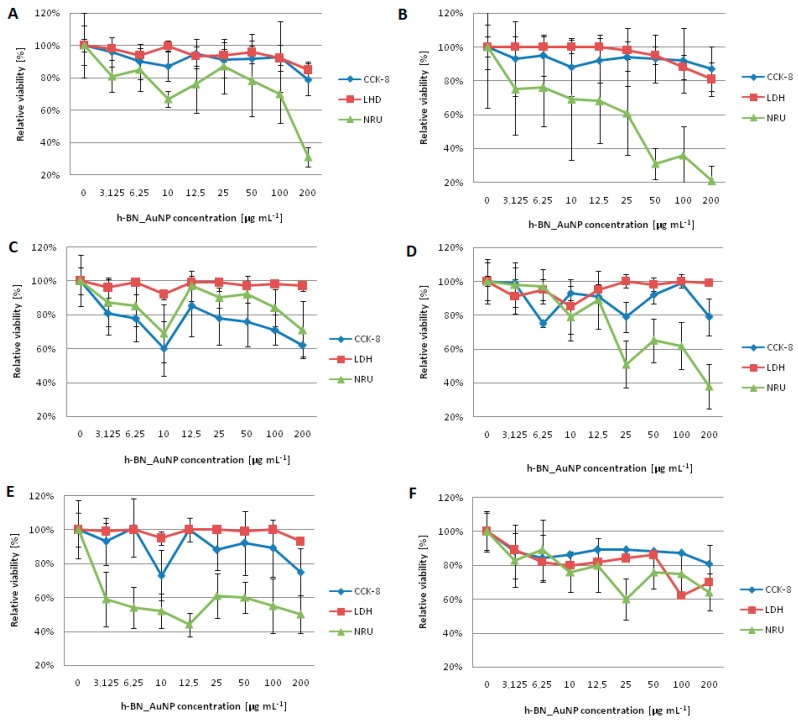
Cytocompatibility analysis based on L929 (24 h—**A**, 48 h—**C**, 72 h—**E**) and MCF-7 cell line (24 h—**B**, 48 h—**D**, 72 h—**F**). Bars represent standard deviation and different small letters (‘a’—for CCK-8 results, ‘b’—for LDH results, ‘c’—for NRU results) indicates statistically significant difference (*p* < 0.05).

Cytocompatibility of the h-BN_Au nanocomposite at 3.125, 6.25, 10.0, 12.5, 25.0, 50.0, 100.0 and 200.0 µg mL^−1^ concentrations was determined using CCK-8, LDH and NRU assays (Figure 8A–F). Both selected cell lines, L929 and MCF-7, exhibited minimal reduction in mitochondrial activity in the CCK-8 assay. The highest reduction of the mitochondrial metabolism was recorded at the concentration of 200.0 µg mL^−1^ for L929 cells incubated for 48 and 72 h (Figure 8C,E). Mitochondrial activity at the concentration of 10.0 µg mL^−1^ and 200.0 µg mL^−1^ was reduced to 75% and 60%, respectively, compared to free-grown L929 cells. In contrast, mitochondrial activity of MCF-7 cells decreased to 80% at a concentration of 200.0 µg mL^−1^ during a 72-h incubation compared to the control cultures (Figure 8F).

The integrity of L929 cell plasma membranes was affected the most by the h-BN_Au nanocomposite in the range of 100.0–200.0 µg mL^−1^ (Figure 8B,F). LDH leakage at 100.0 µg mL^−1^ was increased for L929 cells by 8% in comparison to control samples and by 15% compared to control samples at the 200.0 µg mL^−1^ concentration during the 24-h exposure. L929 cells incubated with the novel nanocomposite for 72 h at the 200.0 µg mL^−1^ concentration exhibited an increase in LDH leakage by 7% compared to the control samples. As presented in Figure 8A–F, h-BN_AuNP did not affect the integrity of L929 cell membranes in a dose-dependent manner. Lactate dehydrogenase leakage was observed in the MCF-7 human breast adenocarcinoma cell line during the cell incubation with the h-BN_Au nanocomposite within the concentration range of 10.0–200.0 µg mL^−1^ (Figure 8B,D,F) and it did not influence the cells in a dose-dependent manner. The highest LDH release was 38% for the 100.0 µg mL^−1^ concentration of tested nanomaterial after a 72-h incubation. The h-BN_Au nanocomposite, at concentrations ranging from 3.125 to 12.5 µg mL^−1^, did not cause any disruption of the cell membrane integrity within 24 h. Longer exposure to the nanocomposite resulted in LDH release.

In contrast to LDH assay results, the neural red uptake (NRU) assay showed different tendency with respect to the viability of both cell lines (Figure 8). As regards the L929 cell line, the relative viability decreased to approximately 45–85% at the 3.125–100.0 µg mL^−1^ h-BN_AuNP concentration range (Figure 8A,C,E). The lowest relative viability was observed when L929 fibroblasts were incubated with h-BN_AuNP at concentrations of 10.0, 12.5 and 200.0 µg mL^−1^. Similarly, the viability of MCF-7 cells was also reduced. The relative viability was reduced to approximately 55–60% in the range of 3.125–25.0 µg mL^−1^ of the nanomaterial, whereas the viability of MCF-7 cells at higher doses of the nanomaterial (50.0–200.0 µg mL^−1^) decreased to 20–60% compared to control cultures (Figure 8B,D,F).

### 3.4. Cellular Uptake Results

**Figure 9:**
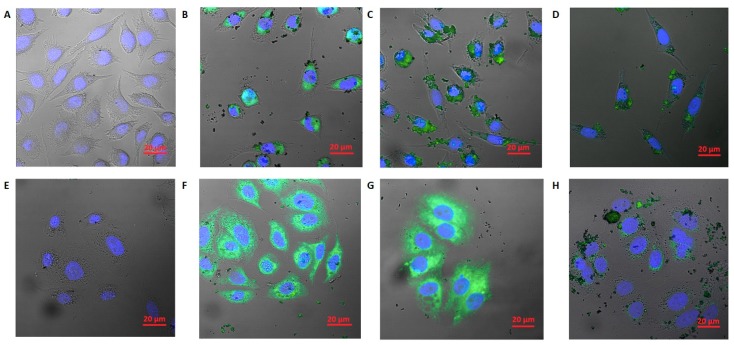
Confocal laser scanning microscopy images. L929 and MCF-7 cells incubated with h-BN labeled with FITC at concentration of 50.0 μg mL^−1^. L929 control culture (**A**), L929 culture at 24 h (**B**), 48 h (**C**) and 72 h (**D**), MCF-7 control culture (**E**), MCF-7 culture at 24 h (**F**), 48 h (**G**) and 72 h (**H**).

The uptake process of hexagonal boron nitride nanoflakes labeled with FITC by normal and cancer cells in three different time points (24, 48 and 72 h) was visualized using confocal microscopy. Figure 9 presenting the intercellular localization of h-BN-FITC in cell cytoplasm—it was confirmed by green fluorescent signal (generated by FITC used for labeling h-BN structures, FT-IR spectrum for the h-BN_FITC was presented in Figure S5, Supplementary materials). The abundance of h-BN was accumulated in the perinuclear region. The presence of h-BN was not confirmed in the nucleus.

## 4. Discussion

### 4.1. The Cytotoxicity of Hexagonal Boron Nitride 

The hexagonal boron nitride (h-BN) compound has a high chemical stability and its colorlessness makes this material convenient for detection of the optical signals derived from DNA, proteins and/or other molecules. On the other hand, h-BN is hardly soluble due to inertness of BN structure, which limits its integrations into biological systems. Thus, many approaches have been developed to solve the problem of BN materials’ solubility (e.g., by functionalization of BN surface with hydroxyl groups, alkyl chains, interactions between BN with guest molecules) [21]. The concentrations for the stabilized BNs are limited. As it was stated by Zhi and co-workes (2005) and Wang et al. (2008), typically, BNs are stable at concentrations lower than the order of 0.01 mg mL^−^^1^ [9,38,39]. For the same reason, the analysis of h-BN impact on living matter (in vitro and in vivo) is undoubtedly important [40]. Lu et al. [40] reported that highly dispersed water-soluble ultrathin h-BN nanoplates (of approx. 30–60 nm in diameter and 16 nm in thickness) did not increase the apoptotic rate of HEK-293T (human embryonic kidney cells 293) and CHO (Chinese hamster ovary) cells in the apoptosis assay. The classical MTT (2-(4,5-dimethyl-2-thiazolyl)-3,5-diphenyl-2H-tetrazolium bromide) assay also confirmed high biocompatibility of h-BN in the concentration range of 0–100.0 μg mL^−1^ after 48-h incubation [40]. Ultrathin hexagonal boron nitride nanoplates were also prepared and tested by Nurunnabi et al. [41]. The exfoliated h-BN was partially functionalized with -OH groups in the latter study and h-BN-OH was analyzed to evaluate the potential cytotoxicity by incubating KB cells with h-BN-OH (at concentrations of 10, 20, 50, 100, 250 and 500 μg mL^−1^) for 24 h. Relative viability of KB cells was measured using the MTT assay. The cytotoxicity of h-BN-OH was not significant even at the highest concentration and cell viability was higher than 90%. In addition, the hemolysis assay demonstrated that h-BN-OH is a blood-compatible material (hemolysis was approximately 4% at the 100 μg mL^−1^ concentration) [41]. 

Weng and co-workers (2014) developed the solid reaction resulting in highly water-soluble and porous BNs. Via thermal substitution of C atoms with boric acid (H₃BO₃) substructures in graphitic carbon nitrides (g-C_3_N_4_), the hydroxylated BN structure with 60% B atoms with hydroxyl groups was obtained. The BN(OH)*_x_* (*x* = 0.6–0.9) displays high hydroxylation degrees and forms stable water solution (2.0 mg mL^−1^) [21]. Novel highly water solubility BNs demonstrated low cytotoxicity (CCK-8 assays showed that more than 92% cells displayed viability after 24 h exposure to 100 μg mL^−1^) and were tested to determine drugs delivery and releasing capability. The human prostate cancerous cells (LNCaP) exhibited dose-dependent sensitivity to the DOX@BN(OH)*_x_* during 24-h exposure. Moreover, the DOX-loaded BN materials exhibited higher cytotoxicity than free doxorubicin [21]. In another study Li et al. (2017) analyzed hollow BN spheres with controlled crystallinity and solubility in two prostate cancer cell cultures—LNCap (androgen-sensitive) and DU-145 (androgen-independent). The fabricated hollow BN spheres were used as a carrier of boric acid (BA) and source of boron (B) that was released by adjusting the temperature. The cells treated with hollow BN spheres and with BN spheres with controlled release of B demonstrated to inhibit the proliferation and apoptosis. caspase-3/7 activity and LDH release confirmed therapeutic property of tested nanomaterials. Furthermore, in vivo analysis in male BALB/c-nu/nu mice demonstrated the suppression of prostate cancer and inhibition of tumor growth [42]. Chan et al. [43] developed binary polypropylene (PP) composites with hexagonal boron nitride and ternary hybrids with h-BN and nanohydroxyapatite (nHA) and tested the obtained materials using osteoblastic cell culture and the MTT assay. Based on the results, Chan et al. [43] concluded that osteoblasts were able to attach to the surface of PP/h-BN, PP/h-BN-nHA and proliferate. The MTT assay showed the cytotoxicity in the rage of 60–75% for 4- and 7-day incubation periods [43]. 

In the studies based on boron nitride nanotubes (BNNTs) it was stated that the up-take mechanism may be depended on the BNNT coating as was demonstrated by Chen and co-workers [25,44,45]. The use of biopolymers (such as PLL, GC or PD) for coating of the BNNTs affected the cellular internalization process. The coated BNNTs were easily up-taken by several cell lines. The fluorescence labeled BNNTs showed that this type of nanomaterial was uptaken after 6–12 h of incubation. The TEM analysis showed that the BNNTs were predominantly located in membrane vesicles [46,47]. In the experiment of Ciofani and co-workers (2014), BNNTs were dispersed and stabilized in aqueous solution by the addition of Arabic gum and the effect of gum-coated ultra-pure boron nitride was evaluated on SH-SY5Y (human neuroblastoma) and HUVEC (human umbilical vein endothelial) cell lines. The BNNTs were well tolerated by both cell lines at the concentrations up to 20 μg mL^−1^. The viability, proliferation, ROS production and apoptosis level were determined and confirmed positive interaction of BNNTs with in vitro models [48]. In our study the uptake of h-BN nanoplates was also confirmed by the visualization of h-BN in cells due to conjugation of nanoplates with FITC. It was found that after 24-h incubation periods nanostructures were present in cellular cytoplasm and perinuclear regions. Other studies on the BNNTs and h-BN as nanocarriers for doxorubicin (Dox) with folate used as targeting agent, were carried out. The Dox loading onto the h-BN was threefold lower than the BNNTs, thus the cytotoxicity study focused only on Dox-BNTTs. It was found that the cellular up-take of folate-Dox-BNNTs was significantly higher than Dox-BNNTs for HeLa cells. The cellular cytotoxicity was determined using HeLa and HUVEC cells. During 8, 24 and 72-h incubation the highest cytotoxic effect was obtained for FA-Dox-BNNTs with significant reduction in cell viability (the viability was in the range of 5 to 40% for HeLa cells and in the range of 20 to 60% for HUVEC). The Dox-BNNTs exhibited cytotoxic effect in the range of 20 to 60% (the same effect was obtained using free Dox) [49]. Feng and co-workes [50] used BN nanospheres (BNNS) conjugated with folate as nanocarriers for doxorubicin (Dox). Obtained BNNS-FA/DOX was recognized and effectively internalized by HeLa cells. It was noticed that HeLa cell proliferation was significantly reduced in the presence of DOX-loaded BNNS. Moreover, BNNS-FA/DOX exhibited higher cytotoxicity than free DOX. Feng and co-workes stated that BNNS-FA complexes are effective drug delivery vehicles [50].

Singh and co-workers [51] tested the biocompatibility of boron nitride in the context of Boron Neutron Therapy (BNCT). The nanostructured BN was added to various cell line cultures: HeLa, HEK-293 and MCF-7 in the concentration range of 0.25–1.0 mg mL^−1^ and incubated for 24 and 48 h to determine the cellular response. The authors observed that cancerous cell lines (HeLa and MCF-7) demonstrated lower relative viability in the MTT assay. Cell survival rate was the lowest after 48-h incubation and reached 40% for MCF-7 and 30% for HeLa cells at the 2 mg mL^−1^ concentration. HEK-293 cells exhibited by 50% higher survival rate during 48-h incubation and by 70% during 24-h exposure at 2 mg mL^−1^ concentration, thus Singh et al. [51] concluded that the cytotoxicity of BN nanostructures was higher for cancerous cells in comparison with normal cell lines [50]. Singh et al. [51] suggested that a higher membrane pore size might be responsible for the enhanced uptake of BN, which led to a higher cytotoxicity, but these conclusions were not validated and need to be evaluated in further investigations [51]. These findings partially confirmed the effect obtained in our study. The analysis of the nanomaterial internalization indicated that it proceeded most efficiently in the first 24 h of incubation and appeared to be more intensive in MCF-7 cells. A significant amount of h-BN_AuNP was accumulated in the cytoplasm of the cells of both lines. Effective internalization mainly translated into the cells’ ability to uptake neutral red, reducing it to 30% for cells of the L929 line and 20% for the MCF-7 line. Higher toxicity was found for 10 μg mL^−1^ for the h-BN-Au_NPs in L929 and MCF-7 cell cultures. This cytotoxicity results combined with the stability study (in this study the nanocomposite was prepared as a solution in the PBS/Pluronic F-127 and the best stability was demonstrated at a concentration of 12.5 μg mL^−1^ and was considered as stable; Figure 6) one can conclude that the 10 μg mL^−1^ concentration forms the stable solution and affects more effectively the mitochondrial cells activity that 25 or 50 μg mL^−1^. We can also conclude that the concentrations of 3.125 and 6.25 μg mL^−1^ were too low to reduce L929 cell relative viability and MCF-7 after 24- and 48-h incubation. In addition, the population doubling time changed slightly over 24 h for the L929 line, while it was extended by about 10 h for MCF-7 cells compared to the control culture. The internalization of the nanomaterial occurred less effectively in the next 24 and 48 h, as a result of aggregate formation in the biological environment (cell culture medium abundant in proteins). The dispersed part of h-BN_Au penetrated inside the cells, while the nanomaterial, in the form of aggregates, was accumulated mainly on the cell surface [52]. Chemical nature of h-BN is also crucial due to the limited solubilization in aqueous solutions. The incubation of cells for 48 h did not affect the DT value for the L929 line, while the population doubling time of MCF-7 cells was longer. A similar observation for the cells incubated with the nanocomposite for a period of 72 h occurred. During 72-h incubation, only the NRU test demonstrated negative effect of the nanocomposite on L929 cells, whereas the MCF-7 line cells showed reduced viability in NRU as well as CCK-8 (tetrazolium-8-[2-(2-methoxy-4-nitrophenyl)-3-(4-nitrophenyl)-5-(2,4-disulfophenyl)-2H-tetrazolium] monosodium salt) and LDH tests. The decrease in relative viability of MCF-7 cells may be related to the increased pore size in the cell membrane and the increased internalization of the nanocomposite (Figure 9).

Although the results of CCK-8 and LDH assays did not indicate a significant cytotoxic effect of h-BN_Au NPs during 24-h incubation, the cell response monitored using the NR uptake assay demonstrated an opposite trend. Neutral red dye uptake is a measure of functional lysosomes, unlike CCK-8 and LDH assays that are based on enzymatic activity. It was reported previously by Weyermann et al. [46] that different cytotoxicity assays can give different or even divergent results depending on the analyzed agent (e.g., drugs, nanomaterials) and the assay employed in the experiment [53]. The tested agent can evoke different cellular responses and engage various intracellular mechanisms (e.g., effect on lysosomes without affecting the cell membrane, impact on specific organelles or general cytotoxicity) [53]. The same tendency was noticed by Fotakis and Timbrell [54] in the study on the comparison of LDH, NR, MTT and protein assays. In some cases (e.g., CdCl_2_ effect on HepG2 and HCT cells), the cytotoxicity could be observed in the NR assay, whereas no cytotoxic effect was found when other assays were employed. These authors also stated that results were dependent on the cell line type, potentially cytotoxic agents, time of exposure and type of assay used in the experiment. In the work of Fotakis and Timbrell, LDH leakage and protein assays were found less sensitive in comparison to NR and MTT assays and considered more suitable in detecting early toxicity [54]. Presented conclusions are consistent with our results of CCK-8, LDH and NR uptake assays. Although CCK-8 and LDH assays showed that h-BN_Au NPs were biocompatible, the Neutral Red uptake assay demonstrated that lysosome function was affected in both cell lines analyzed in three time points. 

Results obtained in different experiments in vitro were confirmed in vivo. The in vivo effect of boron nitride nanomaterial was investigated by Ciofani et al., Wang et al., Liu et al. [55,56,57]. The boron nitride nanotubes (BNNTs) in aqueous solution were stabilized with polymer—glycol chitosan (G-chitosan) at a 1:1 ratio. The prepared solution of BNNTs coated with G-chitosan at the concentration of 1 mg mL^−1^ was injected into the marginal ear vein of rabbits. The physiological parameters were analyzed at intervals of 0, 2, 24 and 72 h. The hematological analyses (e.g., white cell count, red cell count, platelet count, etc.) did not demonstrate significant differences in the experimental and control groups. At 72 h after injection only platelet count was higher in comparison with control group, but recorded values were in the healthy range for rabbits. The biochemical parameters (renal and hepatic) in experimental group were also closely proportional to the values in the control group. This study demonstrated the absence of negative effects not only on blood parameters, but also on liver and kidney functions [48]. In other interesting study, Salvetti et al. [58] tested multiwalled BNNTs on freshwater planarians. The solution of multiwalled BNNTs was prepared with the addition of a 0.1% Arabic gum and the BNNTs coated with Arabic gum (single injection of 100 or 200 μg g^−1^) were injected into the gut of planarians (*Dugesia japonica*). The short-term effect of BNNTs was determined 4 or 24 h after injection. The long-term (chronic) effect analysis was conducted by the injection of BNNTs twice a week for 15 days (total amount of 100 or 200 μg g^−1^). The TEM analysis of intestinal cells did not confirmed any physiological changes in BNNT-treated animals with the respect to the control. The nanomaterial was found inside cytoplasmic vesicles of intestinal phagocytes. The oxidative stress and apoptosis level was determined in control and in groups of animals of acute and chronic exposure to nanomaterial. Data obtained by Salvetti et al. [58] indicated that BNNTs did not induce DNA damage and apoptosis (number of apoptotic cells in BNNT-treated animals in comparison with control group). The effect of BNNTs was also analyzed in the context of planarian stem cells and stem cell progeny by means of the real-time PCR method. The expression of marker genes for proliferating neoblasts, early neoblast progeny and late neoblast progeny, was not affected significantly in the group of BNNT-treated animals. Moreover the effect of BNNTs on regeneration process was also monitored. Both acute and chronic BNNT-treated animals did not exhibit morphological abnormalities and the regeneration processes were not compromise [58].

### 4.2. Presence of Au Nanoparticles on h-BN Nanoplates and Their Impact on Cell Activity and Proliferation Rate 

The recent increasing interest in medical applications of gold nanoparticles encouraged scientists to analyze their potential impact on biological systems. Gold nanoparticles were found to be useful in wound healing and infection prevention. Currently, many studies are concentrated around the anticancer properties of gold nanoparticles. Kamala Priya et al. [59] studied GNPs in MCF 7 cell line. The incubation of cells in the presence of GNPs resulted in an efficient cellular metabolism reduction even at the minimum concentration of 2 μg mL^−1^ [59]. The significance of gold nanoparticles in nanobiotechnology was also evaluated by Rattanata et al. [60]. Gold nanoparticles conjugated with gallic acid (GA) (at concentrations of 30–150 μM) were analyzed in vitro using M213 and M214 cell cultures. These authors found that GNPs-GA complexes inhibited cancer cell proliferation and caused changes in cellular membrane lipids and fatty acids, which resulted in cell death via apoptosis [60]. Geetha et al. [61] tested newly synthesized gold nanoparticles for their anti-leukemic cancer activity (HL-60 cells). The analysis was based on the MTT assay, DNA fragmentation, apoptosis and comet assays. The results obtained by Geetha and co-workers showed HL-60 response to gold nanoparticles treatment that involved DNA fragmentation (gel electrophoresis showed enhanced DNA fragmentation with increasing exposure to gold nanoparticles and longer DNA tail present in the comet assay) and reduction of relative cell viability (in the MTT assay) [61]. Gold nanoparticles (GNPs) are also considered to be an effective delivery platform. Fu et al. [62] loaded Dox directly on GNPs by forming hydrazone (HDox-GNPs) or amide (SDox-GNPs) bonds. Their cytotoxicity and anticancer efficacy were evaluated using U87, HeLa, MCF-7 and A549 cell cultures. HDox-GNPs and SDox-GNPs were tested in a series of concentrations (10, 50, 100, and 500 nM; 1, 5, and 10 μM) for 72 h. mGNPs (monofunctional gold nanoparticles) exhibited low cytotoxicity, whereas HDox-GNPs showed evident cytotoxicity (IC50 for U87 cells was 0.19). SDox-GNPs exhibited much lower cytotoxicity against U87 cells. The difference in IC50 for SDox-GNPs was 2- to 18-fold higher compared to HDox-GNPs [62].

From the chemo-physical point of view, the effect of gold nanoparticles may be the result of their unique and well-known catalytic activities. Those structures supported on metal oxides can catalyze various oxidation reactions by molecular oxygen [63,64]. The size of clusters is an important factor affecting the gold activity. The unique catalytic activity emerges when the size of clusters decreases down to 1-5 nm. The catalytic properties of Au and Au_2_, supported on the hexagonal boron nitride (h-BN) surface or impurity point defects in h-BN absorption and activation of O_2_, can be affected by two different mechanisms. The ability of Au and Au_2_ to activate O_2_ is dependent on the electron pushing mechanism and donor/acceptor mechanism [63,64]. Gao et al. [63] reported that weak interactions of gold particles with h-BN led to binding the stimulation and catalytic activation of O_2_ absorbed on Au/h-BN [63]. Strong absorption of surface defects is related to the charge transfer from/to the adsorbate. That charge transfer can affect the gold catalytic activity. The interaction between Au and the h-BN surface can affect the CO oxidation reaction and oxygen reduction reaction (ORR) by O_2_. The defect-free h-BN surface promotes the electron transfer from Au to O_2_ (pushing electrons from gold to adsorbed oxygen) [65,66].

From a biological standpoint, transition metal nanoparticle were found to induce the chromosomal aberrations, DNA strand breaks, oxidative DNA damage and mutations. DNA single strand breakage can be induced by ${\mathrm{OH}}^{•}$ via formation of 8-hydroxylo-2’-deoxyguanosin (8-OHdG) DNA adduct. In vivo and in vitro experiments demonstrated that NPs, including Cu, Fe, Ti and Ag metal oxides cause micronuclei and DNA damage [67]. Oxygen free radicals, such as superoxide ($O_{2}^{•-}$) and hydroxyl (${\mathrm{OH}}^{•}$) radicals and other reactive species (ROS), e.g., hydrogen peroxide (H_2_O_2_) are mediatiors promoting growth, metabolic or cytostatic effects. Many cellular events are regulated by changes in the redox status [68]. It is known that oxidative stress is responsible for the reduction of the proliferation rate by the inhibition of the transition of cells from the G0 to the G1 phase. As a result, the G1 phase is prolonged, the S phase progresses slower due to DNA synthesis inhibition, cell cycle is inhibited through the restriction point and eventually arrested at the cell cycle checkpoints. Some studies showed that the proliferation rate of normal and cancer cells were decreased in the periods of oxidative stress. Other experiments showed that only tumor cell growth was inhibited in the cell cultures as well as in laboratory animals. The oxidative stress affects the rate of cell proliferation by the inhibition of crucial enzymes (e.g., DNA polymerases and cyclin-dependent kinase) [69].

The response of tumor cells to chronic oxidative stress generated in radiotherapy, photodynamic therapy as well as many chemotherapies, is used in anticancer therapies. Antitumor activity is a relationship between the induction of apoptosis and DNA damage induced by oxygen radicals. Cancer cells may be more sensitive to ROS accumulation in comparison to normal cells [70]. Increased production of ROS that reaches the threshold (incompatible with cell viability) enhances the antioxidant mechanism leading to selective cancer cell death without affecting normal cells [71]. On the other hand, one must take into consideration that the oxidative stress within tumor cells may cause resistance to therapy by increasing cellular expression of P-glycoprotein [72].

## 5. Conclusions

In our preliminary study, hexagonal boron nitride was exfoliated using chemical and physical methods and it was functionalized to obtain nanohybrid of h-BN with Au particles. The nanomaterial was characterized to determine the sample quality. The cytotoxicity of h-BN_Au particles was evaluated and it was found that h-BN_AuNPs did not affect the cellular metabolism (CCK-8 and LDH assays), but it had an impact on the function of lysosomes in both normal and cancer cell lines during 24-h exposition. Longer incubation for 48- and 72-h affected the cell relative viability at the concentration of 10 μg mL^−1^. Moreover, h-BN_Au particles demonstrated inhibition of proliferative activity of the MCF-7 cancer cell line in comparison with normal L929 cell line after 72-h incubation period. These results make the new hybrid nanomaterial an interesting tool not only for anticancer therapy, but also can be used as a platform in biosensor design or in tissue engineering. It is worth emphasizing that the effect of the h-BN_Au nanoparticle on living structures should be examined in greater detail.

## Supplementary Materials

The following are available online at http://www.mdpi.com/2079-4991/8/8/605/s1.
