# Supplementary material for: Hexagonal Boron Nitride Functionalized with Au Nanoparticles—Properties and Potential Biological Applications
Source: Nanomaterials (Basel). 2018 Aug 9;8(8):605. doi: 10.3390/nano8080605 (PMC6116289; doi:10.3390/nano8080605)
Supplement: Supplementary file 1 [file nanomaterials-08-00605-s001.zip › nanomaterials-333456-SI.pdf]

Supporting Information

Article

Hexagonal Boron Nitride Functionalized With Au Nanoparticles –Properties And Potential Biological Applications

Magdalena Jedrzejczak-Silicka[1,*], Martyna Trukawka[2], Mateusz Dudziak[2], Katarzyna Piotrowska[3] and Ewa Mijowska[2,*]

[1] Laboratory of Cytogenetics, West Pomeranian University of Technology, Szczecin, Klemensa Janickiego 29, 71-270 Szczecin, Poland; mjedrzejczak@zut.edu.pl (M.J-S.)

[2] Nanomaterials Physicochemistry Department, West Pomeranian University of Technology, Szczecin, Piastow Avenue 45, 70 – 311 Szczecin, Poland; martyna.brylak@zut.edu.pl (M.T.); mateusz.dudziak@zut.edu.pl (M.D.); emijowska@zut.edu.pl (E.M.)

[3] Department of Physiology, Pomeranian Medical University, Powstancow Wlkp. 72, 70-111 Szczecin, Poland; piot.kata@gmail.com (K.P)

* Correspondence: mjedrzejczak@zut.edu.pl; Tel.: +48-914-496-804; emijowska@zut.edu.pl; Tel.:+48-914-494-742;

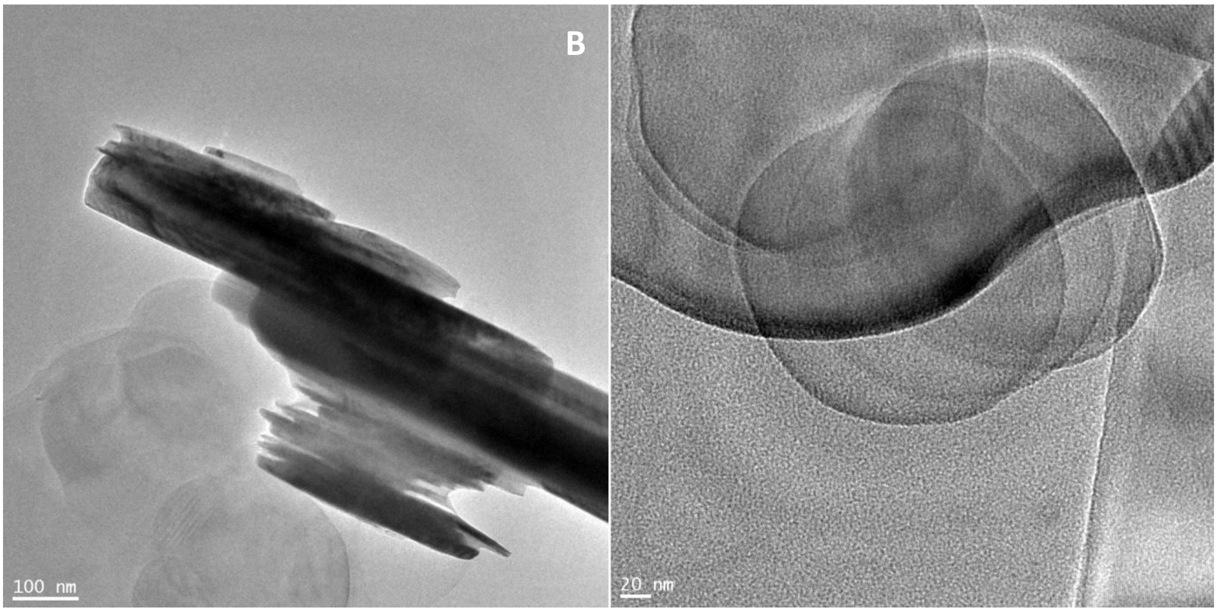

Figure S1. Transmission electron microscopy images of commercial (A) and exfoliated h-BN (B).

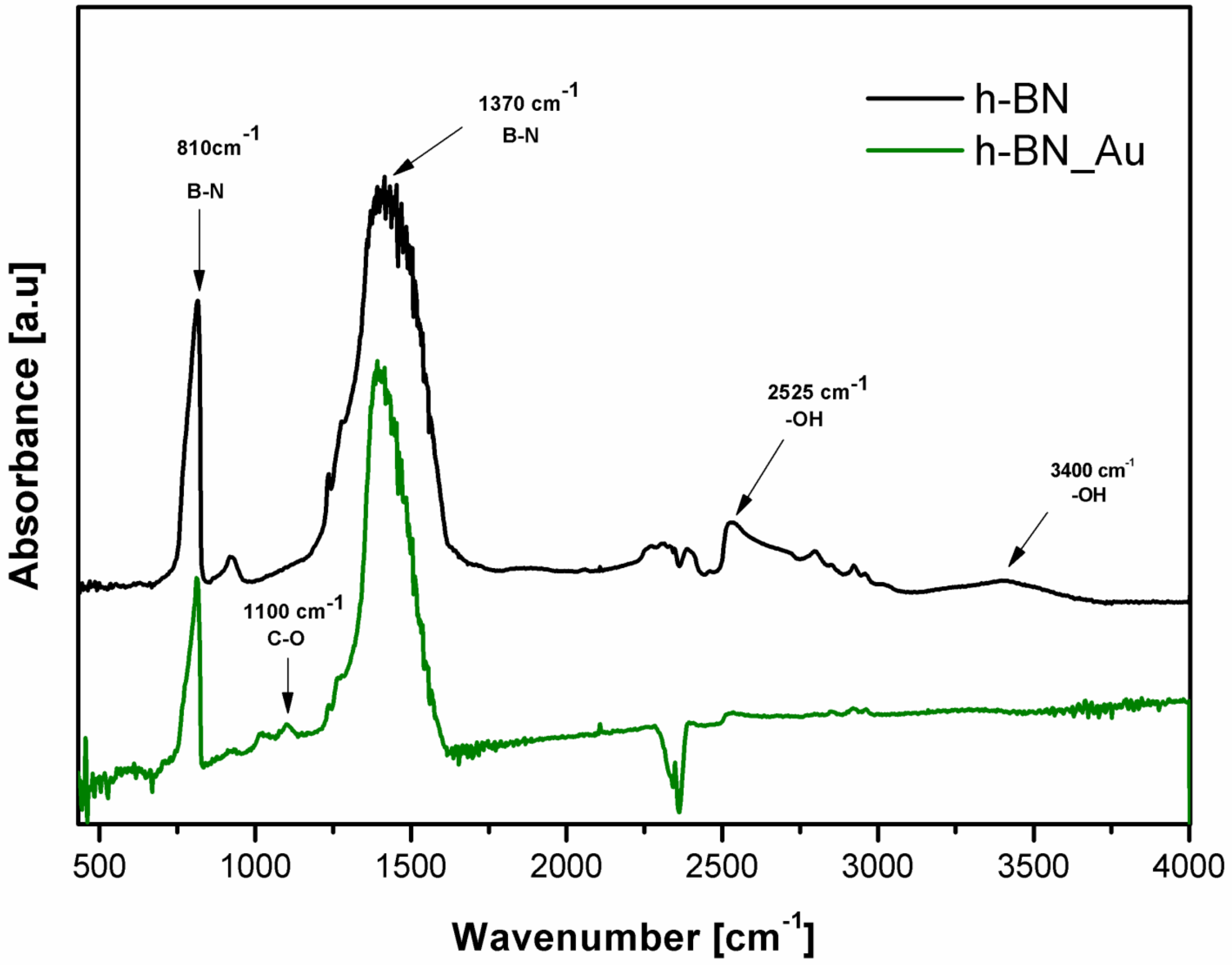

Figure S2. FT-IR spectra of h-BN and h-BN_Au.

A

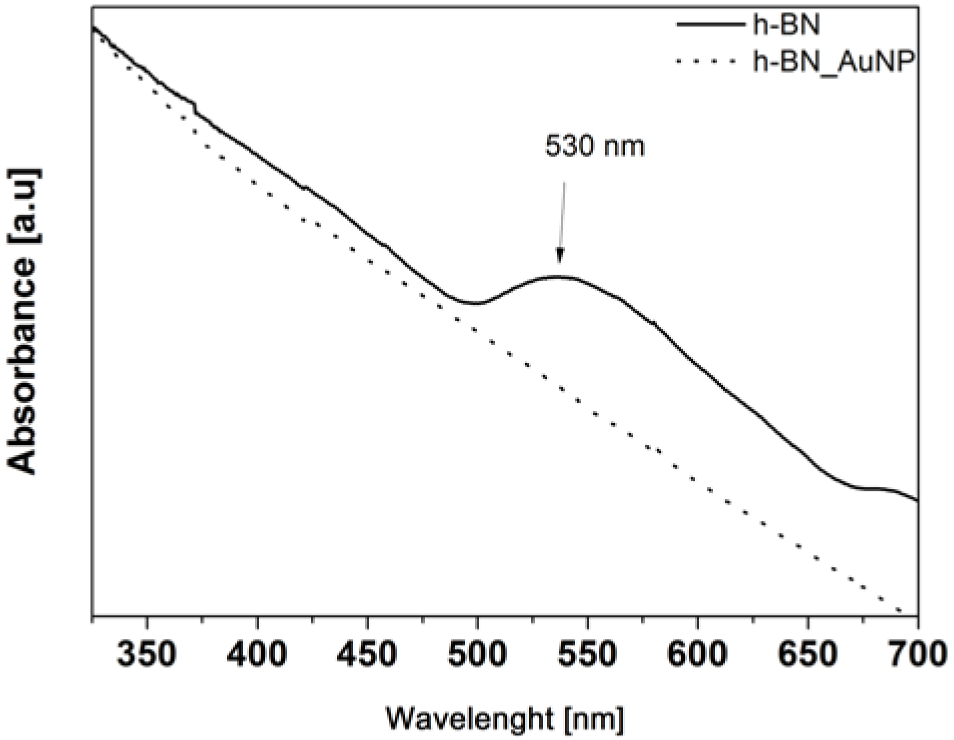

B

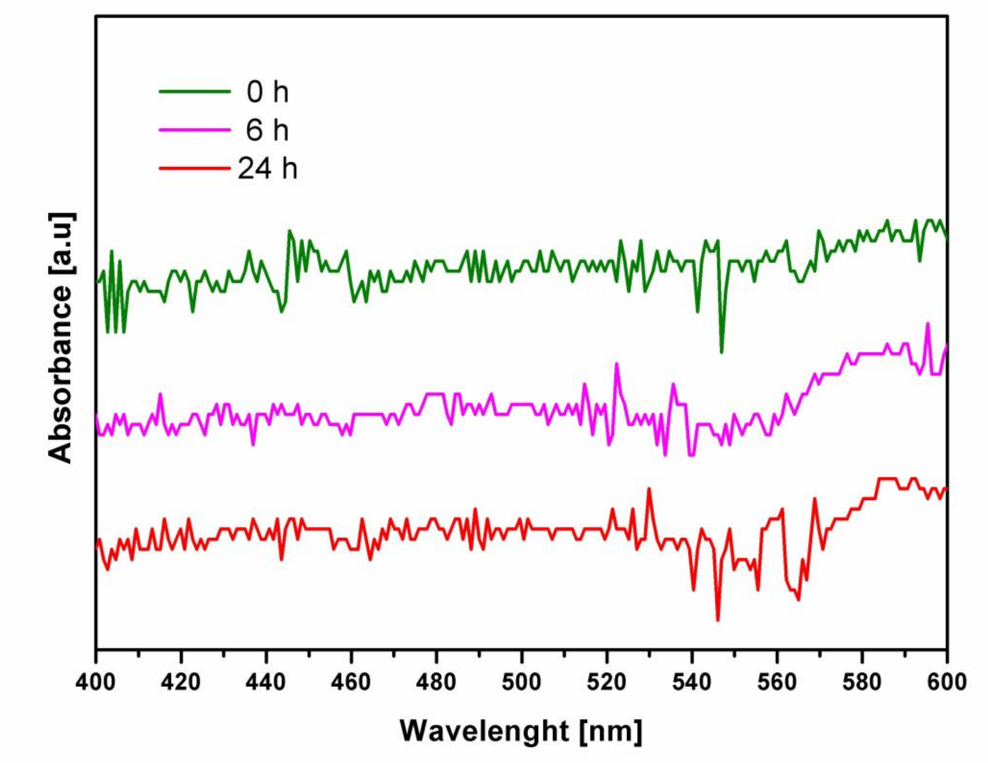

Figure S3. UV-Vis spectra of h-BN and h-BN_AuNP nanocomposite (A) and stability of h-BN and Au nanoparticles connection (B).

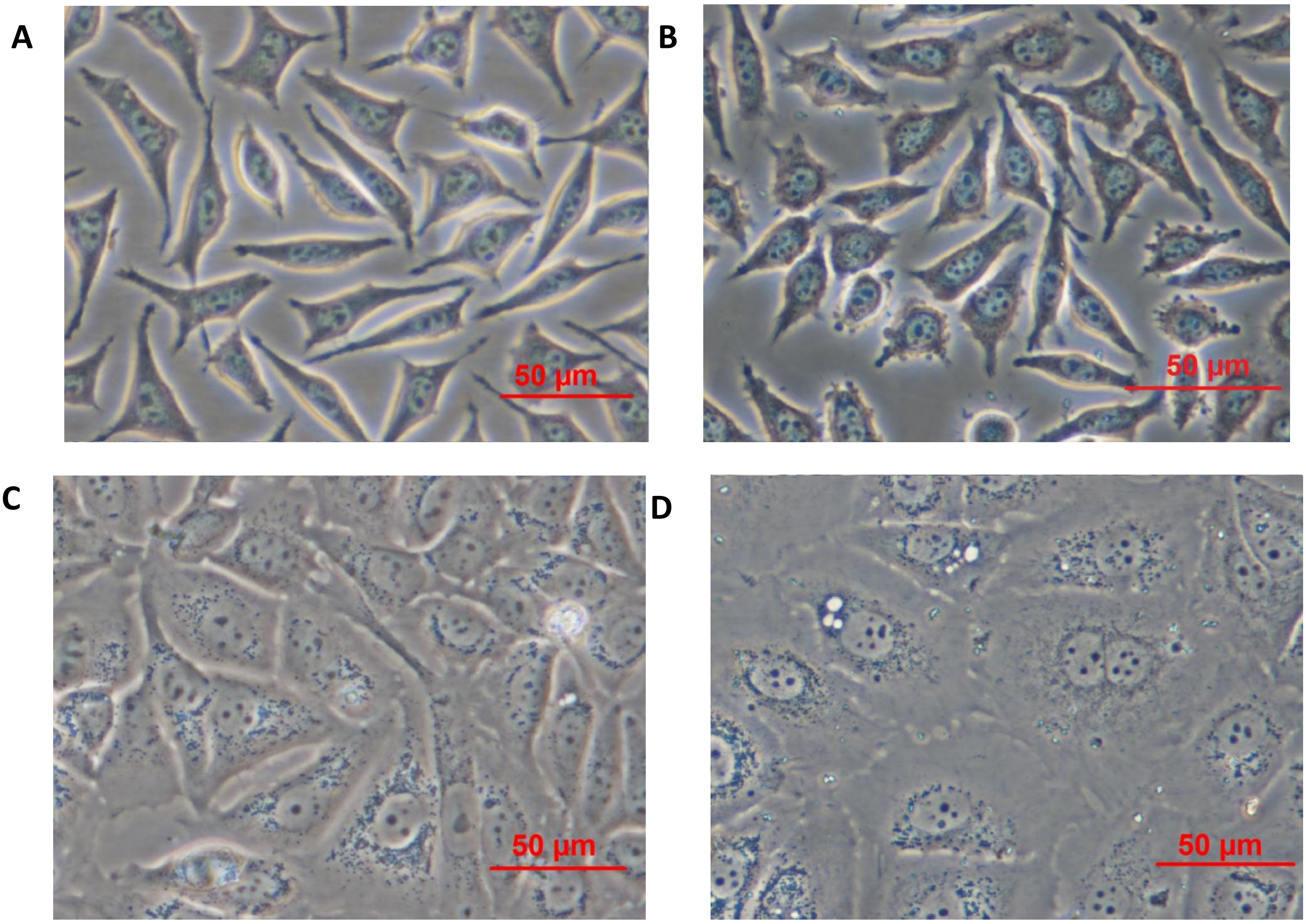

Figure S4. The morphology of L929 and MCF-7 cell lines. The L929 control culture (A), cell culture after incubation with the h-BN_AuNP nanocomposite (B), MCF-7control culture (C) and MCF-7 cells after incubation with the h-BN_AuNP nanocomposite (D).

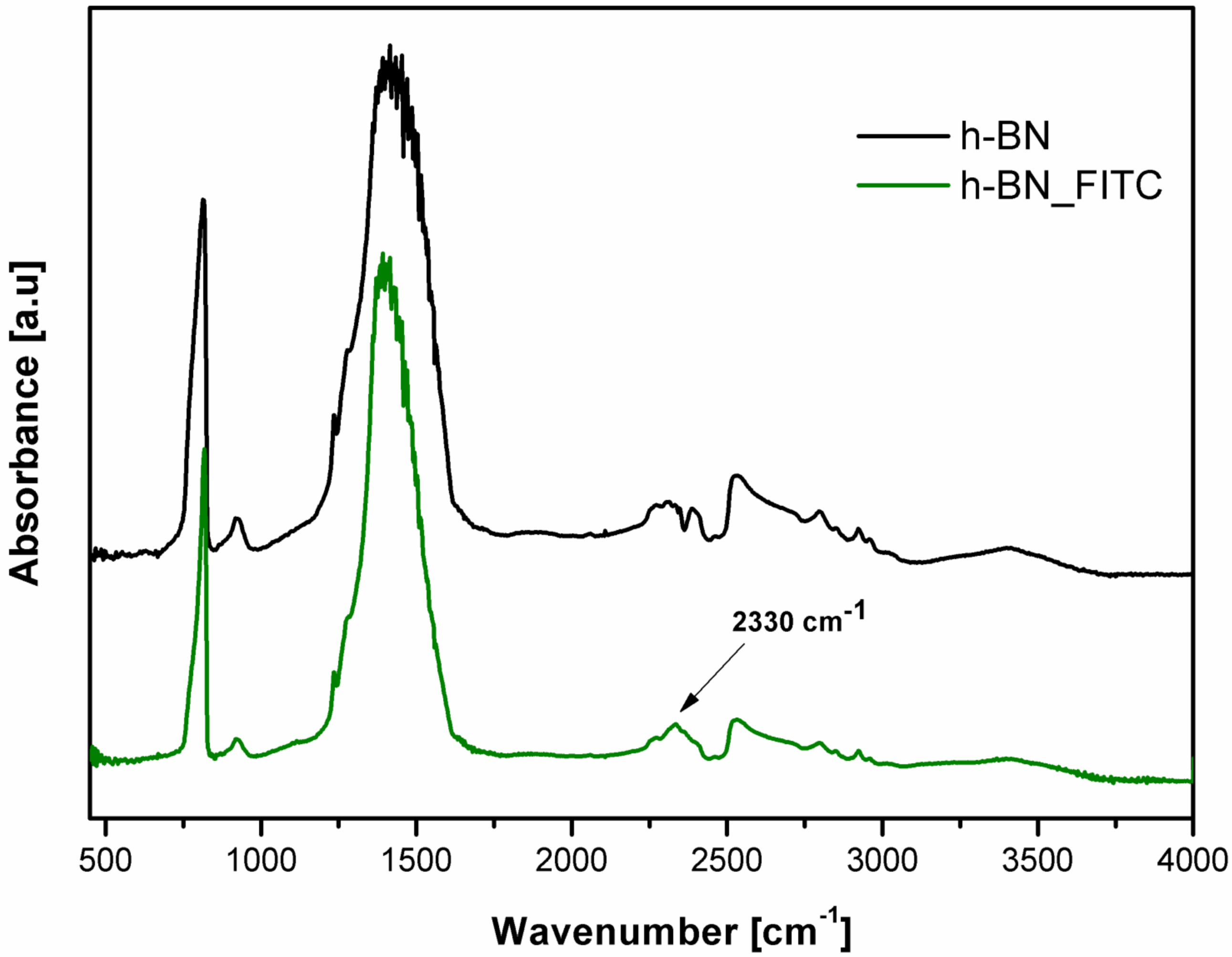

Figure S5. FT-IR spectra of h-BN and h-BN-FITC.
